# Immune Modulation in the Treatment of Amyotrophic Lateral Sclerosis: A Review of Clinical Trials

**DOI:** 10.3389/fneur.2017.00486

**Published:** 2017-09-25

**Authors:** Syed I. Khalid, Leonel Ampie, Ryan Kelly, Shafeeq S. Ladha, Christopher Dardis

**Affiliations:** ^1^Chicago Medical School, Chicago, IL, United States; ^2^Surgical Neurology Branch, NINDS, National Institutes of Health, Bethesda, MD, United States; ^3^Department of Neurological Surgery, University of Virginia School of Medicine, Charlottesville, VA, United States; ^4^Georgetown University School of Medicine, Washington, DC, United States; ^5^Department of Neurology, Barrow Neurological Institute, Phoenix, AZ, United States

**Keywords:** amyotrophic lateral sclerosis, immunotherapy, microglial activation, neuroinflammation, SOD1

## Abstract

Amyotrophic lateral sclerosis (ALS) is a progressive neurodegenerative disease characterized by the degeneration of motor neurons. Though many molecular and genetic causes are thought to serve as predisposing or disease propagating factors, the underlying pathogenesis of the disease is not known. Recent discoveries have demonstrated the presence of inflammation propagating substrates in the central nervous system of patients afflicted with ALS. Over the past decade, this hypothesis has incited an effort to better understand the role of the immune system in ALS and has led to the trial of several potential immune-modulating therapies. Here, we briefly review advances in the role of such therapies. The clinical trials discussed here are currently ongoing or have been concluded at the time of writing.

## Introduction

Amyotrophic lateral sclerosis (ALS) is a progressive neurodegenerative disease characterized by the dysfunction and loss of motor neurons in the brain and/or spinal cord. The striking clinical heterogeneity seen in ALS implies that a single molecular mechanism is unlikely to be responsible for the onset of the disease. The underlying pathophysiology of ALS is not well understood or characterized to date. The presence of pro-inflammatory markers in those demonstrating symptoms has been recognized but the optimal way to modulate this cause of (and response to) neuronal injury remains to be established.

In ALS, cardinal disruptors of cellular homeostasis were originally thought to be oxidative stress and glutamate-mediated excitotoxicity. More recently, additional pathogenic processes have been identified involving protein (metabolism, misfolding, and aggregation), RNA (altered binding), the endoplasmic reticulum and vesicular transport (1, 2). It has also become evident that inflammation plays a crucial role in mediating neuronal injury and disease progression (3, 4). Thus, targeting the immune system would appear to be a more tractable means of slowing the clinical progression of the disease.

Here, we present a brief overview of inflammation in the central nervous system (CNS) as relevant to ALS, particularly microglial homeostasis (5). We then summarize clinical trials to date of immunomodulatory agents based on these recent insights.

The best-studied animal model of ALS replicates a mutation found in familial ALS (fALS), superoxide dismutase 1 (SOD1). However, only 10–15% of ALS is familial and SOD1 mutations account for just 10–12% of fALS, thus just 1–2% of all ALS in humans (6).

The most common genetic cause of fALS has been identified as a six-nucleotide repeat expansion in the *C9orf72* gene (chromosome 9, open-reading frame 72). No single abnormality appears to occur with similar frequency in sALS.

Amyotrophic lateral sclerosis and frontotemporal dementia (FTD) are now often said to be the ends of spectrum, pathologically (7). In the case of ALS-FTD (ALS with FTD), the *C9orf72* expansion appears to account for approximately 40% of familial and 5–10% of sporadic cases (8). This appears to have important effects on microglia, discussed below.

## Immune System in ALS

For much of the twentieth century the CNS was thought to be ‘immune privileged’ i.e. protected from invasion from inflammatory cells. Activity of the immune system within the CNS is now widely accepted.

A lymphatic system lines the dural sinuses and carries immune cells and fluid to cervical lymph nodes (9). Microglia, closely related to the macrophages found in other organs, reside in the CNS. These cells are capable of screening the entire nervous system for foreign material every few hours (10). While circulating lymphocytes do not pass through an intact blood–brain barrier (BBB), extravasation does occur during periods of inflammation (11). In the discussion below, we refer to proteins with their official NCBI names (followed by more common/historical nomenclature) (12).

### Pro- and Anti-inflammatory Immune Phenotypes in ALS

Both microglia (M) and T-cells appear to have central roles in the pathogenesis of ALS. As in other organs, once activated in response to injury or antigen, microglia and helper T-cells (T_h_) differentiate into a pro-inflammatory (classical, M1 and T_h_1) phenotype. Once the inciting event has been dealt with, these cells transition to an anti-inflammatory phenotype (‘alternative’, M2 and T_h_2). This process appears best suited to acute injury; when the pathogenic stimulus cannot be adequately cleared, chronic inflammation develops with persistent M1, T_h_1 activity that can cause unintended injury to local tissues.

**Figure 1:**
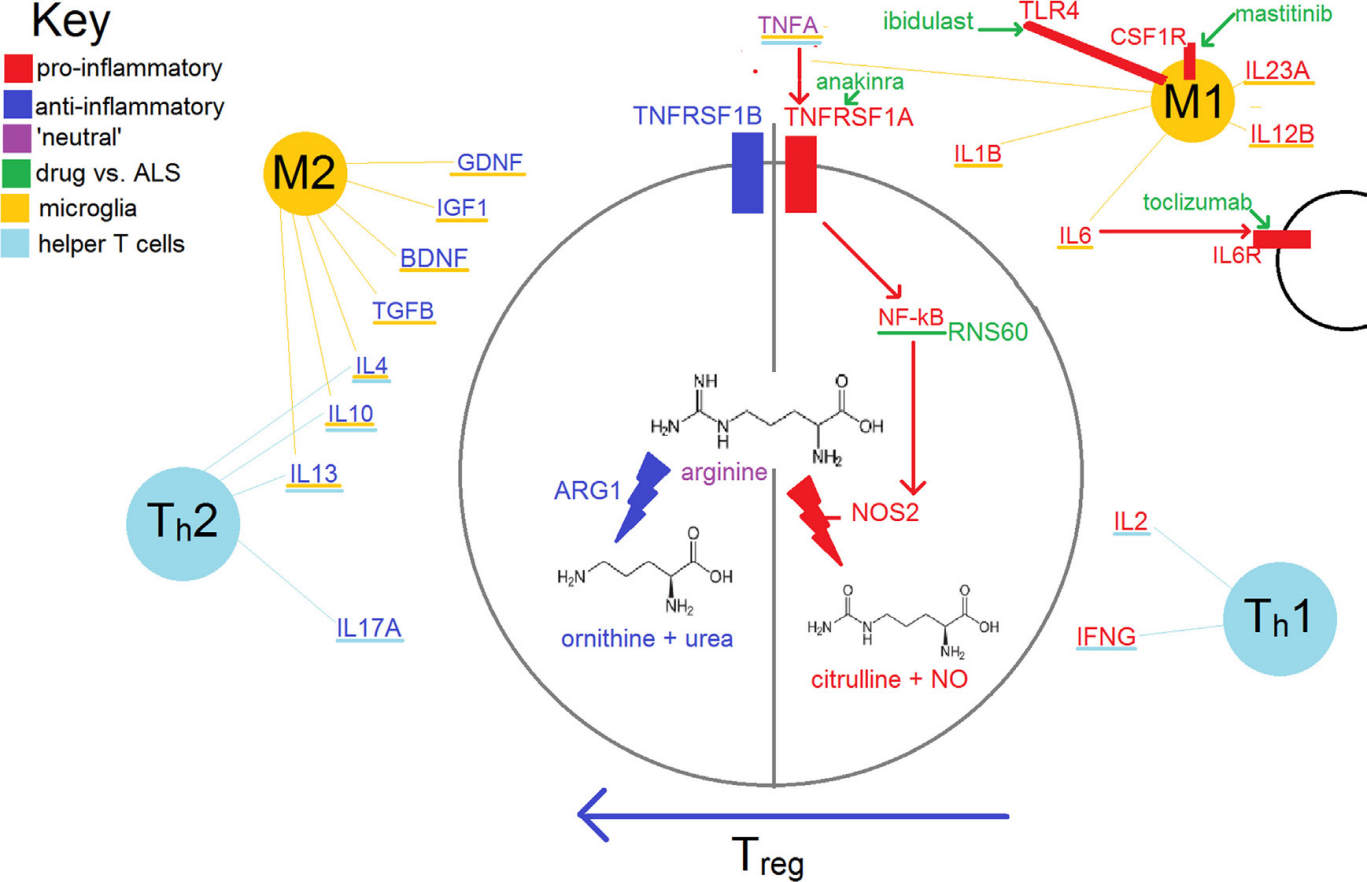
Simplified schema of inflammation in amyotrophic lateral sclerosis (ALS). Some of the treatments in this review with relatively ‘specific’ modes of action are also shown.

A crucial element in ‘tipping the balance’ from one state to the other, particularly evident in microglia, is the relative activity of NOS2 (inducible nitric oxide synthase) vs. ARG1 (arginase 1); this is shown in Figure 1. This balance is particularly important for microglia, but is also used by other elements of the immune system, including T-cells. The expression of NOS2 is increased by transcription factor nuclear factor kappa B (NF-κB), expression of which is increased by binding of tumor necrosis factor alpha (TNFA) to TNF receptor superfamily member 1A (TNFRSF1A). TNFA can also have antiapoptotic effects and lead to an increase in neurotrophic factors *via* TNFRSF1B (discussed further below).

### Microglia

**Figure 2:**
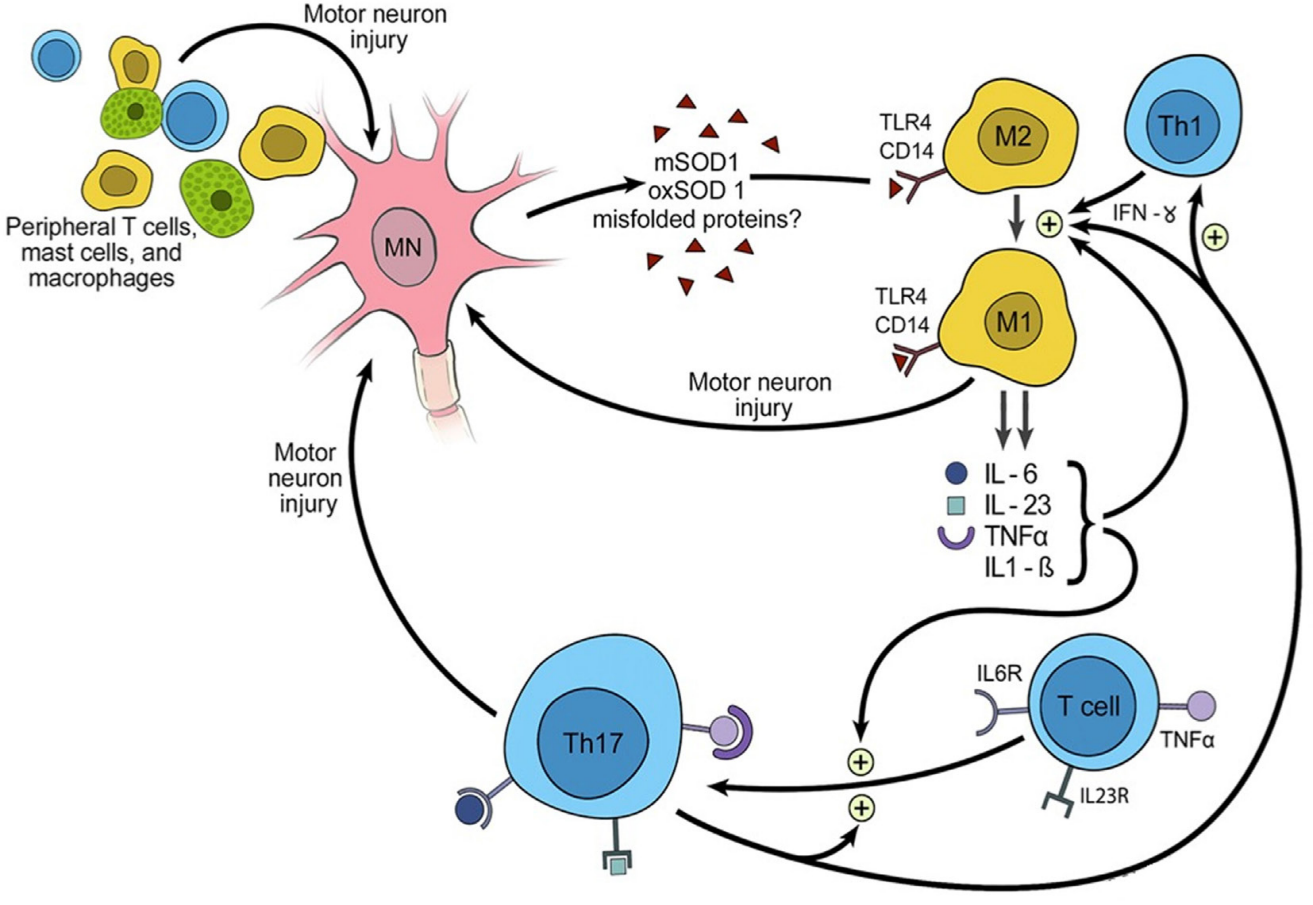
Triggering of inflammation and the role of inflammation in propagating amyotrophic lateral sclerosis.

Like macrophages, microglia become activated *via* the receptor complex CD14 + toll-like receptor 4 (TLR4) in response to antigens such as lipopolysaccharide, proteins released from damaged cells (including SOD1protein aggregates, beta-amyloid and alpha-synuclein), to infiltrative lymphocytes and to signals from the humoral (i.e. antibody-mediated) immune system (Figure 2). Relative to the macrophages of other tissues, microglia are less potent activators of the immune system. This is in part due to their lower expression of protein tyrosine phosphatase, receptor type C (aka CD45), leukocyte common antigen and the major histocompatibility complex (MHC) (13, 14).

The M1 phenotype shows increased expression of pro-inflammatory cytokines such as nitric oxide species, TNFA, and the interleukins (IL) 1B, IL2, and IL6 (4). Receptors and transmembrane proteins involved in antigen presentation are upregulated, including MHC class II, CD86 (B7-2, CTLA-4 counter-receptor B7.2), and the Fc fragment of IgG receptor III (FCGR3; CD16) (15). They also increase production of reactive oxygen species (ROS), which are cytotoxic.

M2 microglia secrete anti-inflammatory cytokines such as IL4, IL10, IL13, TGFB and ARG1 as well as neurotrophic factors such as glial cell line-derived neurotrophic factor, brain-derived neurotrophic factor (BDNF), and insulin-like growth factor-1 (IGF1). IGF1 has the capacity to promote growth and differentiation of ‘neural stem cells’, helping with tissue repair (16, 17). Repair is also mediated *via* angiogenesis and by remodeling of the extracellular matrix (18).

Thus, neurodegeneration is facilitated by the lack of neurotrophic growth factors and by the continued production of cytotoxic byproducts of a pro-inflammatory response.

One factor favoring the inflammatory phenotype (and contributing to neuronal vulnerability to inflammation) is the *C9orf72* expansion. This is in a region which functions as an untranslated promoter in microglia, but *is* translated by neurons. Under normal circumstances, the highest expression levels in the brain are found in microglia. With the expansion, production of C9orf 72 is impaired in microglia, resulting in impairment of endosome/lysosomal trafficking. When attempting phagocytosis, such cells show an increase in ROS and inflammatory cytokines, thought to be due to impairment of phagosome/lysosome fusion (19). In neurons, the transcribed *C9orf72* RNA and the translation of useless dipeptides from the expanded repeat both appear to contribute to susceptibility to inflammation and thus degeneration (8).

### Helper T-Cells

Th1 cells secrete pro-inflammatory cytokines such as interferon gamma (IFNG), IL2 and TNFB (beta); these proteins also promote the M1 phenotype of nearby microglia/macrophages. T_h_2, like M2 cells, secrete the anti-inflammatory cytokines IL4 and IL10; these cytokines also promote the differentiation of microglia/macrophages along M2 lines.

The balance between T_h_1 and T_h_2 is influenced heavily by regulatory T-cells (T_reg_). These are marked by CD4 and IL2RA (IL2 receptor subunit alpha, CD25). Their development and maintenance depend on the transcription factor forkhead box P3 (FOXP3); so-named as mutations in *Drosophila* cause development of head-like structures at each pole instead of the usual fore/hind-gut.

Regulatory T-cells encourage M2 differentiation and impede the activity of cytotoxic T-lymphocytes (T_c_, including natural killer T-cells). Lower circulating levels of T_regs_ have been shown in those with ALS vs. controls (20). Lower levels of T_regs_ and of FOXP3 have also been correlated with the rate of clinical progression of ALS and may be a useful prognostic biomarker that could be used to stratify groups in clinical trials (21).

## Clinical Trials

### Search Methods

We searched the NIH’s ClinicalTrials.gov (NCT), a database of medical studies in human volunteers, with the term “amyotrophic lateral sclerosis” for any trials focused on modifying inflammation. We included trials currently in progress or completed prior to the time of writing. These trials are summarized in Table 1, where they appear in the same order as the treatments discussed below. We classified these treatments based on their use in other pro-inflammatory diseases. They are sorted (broadly) by treatment and by the year the trial began accrual. Some trials are discussed here which were not registered with NCT. Also, some NCT-registered trials do not yet have a corresponding article reporting results and are referenced here with their NCT identifier.

**Table 1:** Trials of immune-modulating treatments in ALS registered with NCT.

NCT ID	SD	Agent	ra	n	wks	P	RCT	Outcomes		C	R
								Primary	Secondary		
**Rheumatoid arthritis**											
01277315	2/11	Anakinra	po	18	4	2	N	S/T	S/T	Y	(26)
02588677	4/13	Masitinib + riluzole	po	394	48	2/3	Y	ALSFRS-R	VC, QoL	Y	NA
02469896	11/15	Tocilizumab	iv	24	16	2	Y	S/T	ALSFRS-R, VC	N	NA
**Multiple sclerosis**											
00326625	7/06	Glatiramer acetate	sc	366	52	2	Y	ALSFRS-R	ttDV	Y	(38)
01786174	8/13	Fingolimod	po	30	8	2	Y	ALSFRS-R, VC, FEV1	T-cell subsets	Y	(47)
02238626	8/14	Ibudilast	po	120	26	1b/2a	Y	S/T, ALSFRS-R	VC, MMT, HHD	N	(51)
02525471	10/15	RNS60 (saline)	iv	18	24	1	N	S/T	ALSFRS-R, VC, HHD	N	NA
**Anti-inflammatory**											
00355576	7/06	Celecoxib + creatine	po	86	26	2	N	ALSFRS-R	VC, QoL, TGUG	Y	(59)
**‘ALS-specific’**											
01753076	12/12	Ozanezumab	iv	304	48	2	Y	ALSFRS-R, OS	VC, HHD, QoL	Y	(63)
01091142	7/10	NP001 (chlorite)	iv	32	26	1	N	S/T	Biomarkers	Y	(66)
01281631	2/11	NP001	iv	136	39	2	Y	ALSFRS-R	VC, TTT, serum IM	Y	(67)
02794857	8/16	NP001	iv	120	26	2	Y	ALSFRS-R	TTT, serum IM	N	NA
**Treatment of cancer**											
00140452	2/05	Thalidomide	po	18	39	2	N	ALSFRS	S/T, QoL, serum IM	Y	(69)
01257581	3/11	Tamoxifen	po	60	42	2	N	ALSFRS-R	VC, TTT, HHD, ATLIS	Y	(73)
00397423	12/06	Granulocyte colony-stimulating factor (G-CSF)	sc	40	52	2	Y	ALSFRS	AARS	Y	(75)
01825551	11/12	G-CSF	sc	40	13	2/3	Y	ALSFRS-R	CMAP, MMT, ALSAQ-40	Y	(76)
03085706	10/10	Peripheral blood mononuclear cell autotransplantation	sa	14	12	1/2	N	S	Functional independence, balance, dysarthria	Y	NA
02286011	11/14	Bone-marrow mononuclear cells	im	20	104	1	N	S	MUNE, CMAP, MRC	N	NA
**Transplant rejection**											
01884571	10/13	Basiliximab, mycophenolate, tacrolimus, methylprednisolone, prednisone	iv, po	33	52	2	N	ALSFRS-R	VC, HHD, serum IM, CSF IM	Y	NA

*ALS, amyotrophic lateral sclerosis; NCT ID, NIH Clinical Trials identifier; SD, start date (month/year); ra, route of administration; po, oral; iv, intravenous; sc, subcutaneous; sa, subarachnoid space (i.e., into CSF); im, intramuscular; n, number of subjects; wks, duration of trial (weeks); P, Phase (1–3); RCT, randomized, double-blind, placebo-controlled trial? C, completed? (at time of writing); R, reference; NA, not applicable (no results reported yet)*.

*Outcomes—note not all secondary outcomes are listed, for reasons of space*.

*S, safety; T, tolerability; ALSFRS-R, ALS Functional Rating Scale (revised); VC, vital capacity (any method); FEV1, forced expiratory volume in 1 sec; OS, overall survival; QoL, quality of life; ttDV, time to death or ventillator-dependence; MMT, manual muscle testing; HHD, hand-held dynamometry (muscle strength); TGUG, timed get-up-and-go (sitting to walking); TTT, time to tracheostomy; IM, inflammatory markers; ATLIS, accurate test of limb isometric strength; AARS, Appel ALS Rating Scale; CMAP, compound muscle action potential; ALSAQ-40, ALS Assessment Questionnaire-40; MUNE, motor units estimate (number of motor units); MRC, Medical Research Council (score, muscle strength)*.

### Measures of Outcome

The most commonly used measure of outcome in these trials is the change in the ALS Functional Rating Scale (ALSFRS) over time. In 1999, the scale was revised to include assessments of dyspnea, orthopnea, and the need for ventilatory support. This latter, the ALSFRS (revised) (ALSFRS-R) has been adopted by the majority of clinical investigators since (22). Due to the restrictive effect of ALS on pulmonary function, forced vital capacity (FVC) is also often used as a measure of disease progression.

## Drugs Used for Rheumatoid Arthritis

Rheumatoid arthritis (RA) has been noted with a greater frequency than would be expected by chance in the families of those affected by ALS (23). In the case of the individual patient, just seven cases of co-occurring ALS and RA have been reported to date. It is thought that most if not all cases were the result of a chance association; indeed the coincidence of the two conditions would appear to be *less* than might be expected by chance alone.

Notably, as reported by Padovan et al., in two patients with RA, ALS developed rapidly after the introduction of or increase in dose of infliximab, a TNFA antagonist (24). While these authors acknowledge the limitations of such anecdotal observations, this finding would fit with an upregulation of the antiapoptotic TNFRSF1B in chronic RA, thereby masking a tendency toward ALS in the cases they report. Another case in support of this mechanism is that of a patient with psoriatic arthritis who developed ALS rapidly after starting adalimumab, another antibody targeting TNFA (25).

### Anakinra

Interleukin 1 is an endogenous pyrogen and a mediator of autoimmune and infectious diseases (26). Anakinra, a recombinant analog of the endogenous antagonist IL1Ra, has already proven effective in the treatment of refractory RA (27). IL1R antagonists have been shown to extend lifespan in SOD1-G93A mice (28). A pilot study of 17 patients treated with anakinra (at the same dose used for RA, 100 mg daily) for 1 year showed no significant reduction in disease progression, measured with the ALSFRS-R. Though the study did show a decrease in cytokines and fibrinogen during the initial 24 weeks of treatment, there was a ‘rebound’ increase in inflammatory markers during the latter half of the study (29).

### Masitinib

Masitinib is a pluripotent tyrosine kinase inhibitor which affects multiple pro-inflammatory mast cell receptors. It inhibits mast cell degranulation and mobility. Studies of masitinib in RA, systemic mastocytosis, mast cell tumors and in Alzheimer’s disease have already shown promising results. In SOD1-G93A rats, masitinib was shown to decrease microglial cell activation *via* inhibition of colony-stimulating factor 1 receptor (30). Masitinib may also prevent macrophage infiltration into the ventral root by inhibiting KIT (KIT proto-oncogene receptor tyrosine kinase), thereby delaying the ‘dying back’ phenomenon associated with anterior horn cell dysfunction (unpublished data, Emilianos Trias et al.).

A Phase 3 randomized, double-blind, placebo-controlled trial (RDBPCT) is ongoing to determine the efficacy of riluzole plus masitinib vs. placebo in ALS over the course of 48 weeks. The interim results are encouraging, with 50% (191 of 381) of patients having completed the riluzole and masitinib arm. A statistically significant difference was seen in the ALSFRS-R score as well as the secondary end points: change in the Combined Assessment of Function scale and FVC.

### Tocilizumab

Interleukin 6 signaling is major inducer of the acute-phase response; its receptor is blocked by the monoclonal antibody tocilizumab. Increased serum IL6 is recognized in autoimmune diseases such as RA, systemic-onset juvenile chronic arthritis and psoriasis as well as patients with sALS. Using peripheral blood mononuclear cells (PBMCs i.e. T and B cells as well as monocytes) from four patients with sALS, tocilizumab was shown to inhibit the pro-inflammatory effects of adding mutant SOD1-G93A (31). In particular, the expression of a number of cytokines was reduced *in vitro*: IL6, IL1B, TNFA, IFNG and GM-CSF. A Phase 2 RDBPCT investigating the safety and tolerability in adult patients with ALS is ongoing. In addition to safety measures, the study investigates the effect of tocilizumab on novel biomarkers including PBMC gene expression profiles and MRI-PET imaging of activated microglia.

## Drugs Used for Multiple Sclerosis (MS)

Like RA and ALS, MS and ALS appear to aggregate within families, while co-occurrence in one individual appears rare. We are aware of just four such case reports to date (32). Microglial activation appears partly responsible for neuronal injury in both ALS and MS (33).

### Glatiramer Acetate (GA)

Glatiramer acetate is comprised of random polymers of glutamic acid (G), lysine (L), alanine (A), and tyrosine (T). It has long been used in the treatment of relapsing–remitting MS. In such patients, treatment with GA leads to an increase in IL10 and IL4 and a decrease in TNFA (34). GA may also increase T_reg_ activity.

The safety of GA in ALS has been demonstrated in a Phase 1 trial of 20 patients and 10 controls, with doses of 20 mg given daily or biweekly to 10 patients each. GA appeared safe and encouraging immunological changes were measured (35).

A Phase 2 RDBPCT with 366 patients used GA 40 mg/day (vs. ×3/week, as is typical in MS). They remained under observation for 52 weeks. No significant difference in the rate of deterioration (ALSFRS-R) or overall survival was seen (36). The authors speculate that differences in BBB permeability between MS and ALS (affecting the pharmacokinetics of GA or the traffic of the immune system into the CNS) may account for these results.

### Fingolimod

Fingolimod is an inhibitor of the sphingosine 1-phosphate receptor (S1PR). This receptor is found on immune cells, neural cells, endothelial cells and smooth muscle. It is thought to play a role in angiogenesis, neurogenesis and immune regulation/trafficking. Inhibition of S1PR causes sequestration of lymphocytes within lymph nodes, thereby reducing their numbers in the blood stream. It has already proved to be effective in MS (37).

In SOD1-G93A mice, fingolimod improved survival. Its immunomodulatory effects are mediated through NOS2, IL1B, FOXP3, IL10, ARG1, integrin subunit alpha M (CD11B, part of complement receptor 3) and BDNF (38). An RBDPCT, with 2:1 randomization, in 30 patients with ALS showed the treatment to be safe and tolerable (one patient stopped treatment due to QT prolongation) (39). We are not aware of plans to study this agent further in ALS.

### Ibudilast

Ibudilast is a an inhibitor of TLR4 and phosphodiesterase 3 and 4 which has shown immunomodulatory effects in MS by shifting the immune response from T_h_1 to T_h_2 (40). In ALS, TLR4 facilitates the transformation of M2 to M1 microglia (41). The safety profile of ibudilast has been established through long use in the treatment of asthma in Japan. It has been shown to reduce the loss of brain volume change in patients with MS (42). A Phase 2 trial of safety and efficacy in patients with ALS has completed accrual and preliminary reports on safety and tolerability are encouraging (43).

### RNS60

RNS60 is modified saline; it is mixed with oxygen in a controlled manner to generate stable, electrically charged nanobubbles. This agent is undergoing clinical trials in MS as well as ALS. The electrical charge carried by RNS60 affects membrane ion channels and increases mitochondrial ATP production in cell culture (44, 45). In a mouse model of MS, it has been shown to reduce NO and NOS2 production and thus is anti-inflammatory (46). This effect is thought to be mediated through the inhibition of NF-κB (47). This results in ‘tipping the balance’ toward the alternative (M2, T_h_2) response.

As the drug’s effects are a result of biological and not chemical activity, preclinical toxicology studies have shown almost no side effects. Safety has been established in three Phase 1 studies, one intravenous and two using inhalation. A Phase 1 trial of intravenous RNS60 has completed patient accrual (NCT02525471). This will investigate its efficacy as measured by the ALSFRS-R and on inflammatory biomarkers.

## ‘Non-Specific’ Anti-Inflammatory Agents

### Intravenous Immunoglobulin (ivIg)

Two small ‘pilot’ studies to assess the effects of a 3-month course of ivIg on the severity of ALS have proved disappointing; this appears to have put an end to investigation of this modality. These studies were performed prior to the advent of the ALSFRS; instead, participants served as their own controls.

The first in nine patients with ALS used ivIg 2 g/kg monthly for 3 months. No change in disease severity was seen using the outcome measure of maximum voluntary isometric contraction (MVIC), as per the Tufts Quantitative Neuromuscular Evaluation system (48). A subsequent study used the same dose and timing in combination with cyclophosphamide 1–2 mg/kg/day in seven patients over 4–13 months. The rate of deterioration was no slower after starting treatment than before, as measured by the means of the Medical Research Council (MRC) score for muscle strength (10 muscles/limb = 40 muscles), a clinical scale for bulbar function (range 1–5) and the modified Rankin disability scale (range 0–5).

### Celecoxib

Celecoxib, a cyclooxygenase-2 inhibitor (prostaglandin G/H synthase) is used to treat pain and inflammation; it also decreases the prostaglandin-induced release of glutamate. One RDBPCT enrolled 30 patients with ALS and randomized their treatment (2:1) between celecoxib and placebo for 12 months. No difference was found in disease progression (ALSFRS-R), muscle strength (maximum MVIC), FCV, or in estimates of numbers of motor units (49).

Celecoxib has also been evaluated in a Phase 2 adaptive trial. Here, it was compared with minocycline; both were given with creatine in order to aid mitochondrial function (50). Historical controls were used as a comparison. Planned enrollment was with sequential pools of 60 patients per arm; after one pool per arm, the celecoxib–creatine regimen proved superior to that of minocycline–creatine, although not greatly superior to the historical controls, as assessed by the rate of decline in the ALSFRS-R at 6 months (35).

## Agents ‘Specific’ to ALS

### Ozanezumab

Activation of the reticulon 4 receptor (RTN4R, aka Nogo receptor) inhibits the growth of axons in mammals following peripheral nervous system injury. RTN4R is also found on macrophages, where, following Wallerian degeneration, it has been shown to mediate the clearance of these cells from the site of injury (51). Additionally, RNT4 (Nogo-A) is overexpressed in the skeletal muscle of patients with ALS (52).

Thus, ozanezumab (an antibody targeting RNT4) may help slow disease progression, either by decreasing the inhibition of axonal growth or by affecting the inflammatory process following neuronal injury. An RDBPCT with 303 patients tested this treatment, given over 48 weeks. No difference in disease progression (ALSFRS-R) or in survival was seen (53). The results are not thought to have been due to inadequate dosing or pharmacokinetic factors.

### NP001

Taurine is an amino acid not incorporated into protein, which binds HOCl, as produced by the oxidative burst of activated neutrophils. The product of this reaction, taurine chloramine, is a less toxic oxidizing agent than HOCl; it is also a signaling molecule that contributes to the self-limiting nature of inflammation. Sodium chlorite (NaClO_2_; in solution with 63 mM chlorite and pH-corrected = NP001, aka WF10) was developed to mimic this effect. In PBMCs stimulated by anti-CD3 (part of the T-cell receptor complex), WF10 reduced IL2 production as well as the nuclear translocation of the transcription factor nuclear factor of activated T-cells 1 (NFATC1).

WF10/NP001 was originally developed for advanced-stage HIV, to suppress the chronic activation of macrophages, which is thought to be partially responsible for neurological injuries in HIV (54, 55). It is also being investigated as a treatment for MS, although work in ALS is farther along than in these other conditions.

A Phase 1 trial in 32 patients showed the treatment to be safe and well tolerated (56). There was a dose-dependent decrease in a marker of monocyte activation, FCGR3. A subsequent RDBPCT with 136 patients showed no effect on ALSFRS-R, although a subset of those with high baseline serum inflammatory markers (including IL6, IFNG and CRP) did show stabilization of disease (57). A follow-up, focused on those with ALS and high baseline CRP, is ongoing.

## Treatments Used Primarily in Patients with Cancer

### Thalidomide

Thalidomide is an immunomodulating agent whose effects on TNFA have been shown to be beneficial in SOD1-G93A mice (58). A trial using 400 mg/day, with outcomes available in 18 patients, showed no improvement in ALSFRS or FCV vs. historical controls. (This dose is similar to that used for refractory chronic graft-versus-host disease and to that used in the multi-agent treatment of multiple myeloma.) Treatment with thalidomide was associated with a number of adverse effects, including deep vein thrombosis and bradycardia (59). Bradycardia appears common, with another study reporting a rate of 50% in 18 patients, when used in combination with riluzole (60).

### Tamoxifen

A competitive inhibitor of estrogen receptors, tamoxifen continues to be used as adjuvant treatment for breast cancer as well as endometrial carcinoma. Slowing of ALS progression was reported in a woman who started tamoxifen treatment for breast cancer at the 2004 International ALS/MND Symposium. Its anti-inflammatory effect may result from inhibition of protein kinase C (3). A Phase 2 trial in 60 patients reported an increase in survival in those using more than 20 mg/day vs. lower doses (61).

Another Phase 2 trial randomized patients to daily creatine 30 mg or tamoxifen 40 vs. 80 mg. In those taking the higher dose of tamoxifen, disease progression was reduced (ALSFRS-R), an effect which remained significant after controlling for gender, site of disease onset and VC. These results were presented by Atassi et al. at the Northeast ALS Consortium Webinar 2013.^1^

### Granulocyte Colony-Stimulating Factor (G-CSF, Filgrastim)

This compound stimulates the proliferation and differentiation of granulocytes; it has been shown to play a part in neuro- and angio-genesis as well as modulation of the immune system. It is commonly used in myelotoxic chemotherapy to prevent or treat neutropenia. In SOD1-G93A mice, use of pegylated G-CSF increased survival and reduced microgliosis (62).

A pilot study, with 10 patients available for complete assessment, used a dose of 2 g/kg for 5 days. While the first 3 months of showed a significant reduction in the rate of decline (ALSFRS), there was a ‘rebound’ worsening in the subsequent 3 months (63). The decline in compound muscle action potential (CMAP) was also reduced during the first 3 months and remained unchanged thereafter.

An RDBPCT in 10 patients used a dose of 10 μg/kg on days 1–10 and 20–25 (64). No difference in disease progression at 100 days (ALSFRS) was noted. There was a smaller decline in fractional anisotropy of the brain in the treated group. This MRI finding, which measures the ‘directionality’ of water flow, is considered a marker of axonal myelination (65).

Based on these encouraging results, a larger RDBPCT was performed with 40 patients, using G-CSF at 5 μg/kg/q12h for 5 days; they were observed for 3 months. There was no change in disease progression, as measured with the ALSFRS-R, the ALS Assessment Questionnaire-40, manual muscle testing, and CMAPs (66). Although the duration of the trial was relatively short, the authors note “previous studies suggest that a longer duration of follow-up is unlikely to confer any important clinical benefit with currently administered doses of G-CSF.” The small sample size was based on a large expected difference in the change in ALSFRS-R, which may in retrospect have been a little optimistic. Another trial of G-CSF in 40 patients appears to have been completed, although no results are available (NCT00397423).

### White Blood Cell Support

Isolation of mononuclear cells from blood and bone marrow has long been used to provide support for patients undergoing myelotoxic chemotherapy or marrow transplantation. Thus, such facilities are likely to be available wherever such treatments are used.

A single-center, Phase 1 RDBPCT to assess the rate of adverse events related to the intramuscular infusion of autologous mononuclear cells of bone marrow is currently underway and scheduled to complete at the end of 2017 (NCT02286011). A similar study was completed to access the deliverance of PBMC transplantation into the subarachnoid space, but has not yet reported results (NCT03085706).

Another Phase 1 trial with three patients used leukapheresis followed by isolation and expansion of T_regs_. They received four infusions of these T_regs_ every 2 weeks, with simultaneous subcutaneous IL2 (to expand T-cells). This modality appears safe and clinical benefits were noted for up to 1 month after the infusions were stopped (67).

## Treatments Used as Part of Organ Transplantation

### Cyclosporine

Cyclosporine, which reduces production of IL2, is an oral agent which does not appear to cross the intact BBB. When infused into the lateral cerebral ventricle of SOD1-G93A mice, cyclosporine leads to improved survival (68). An RDBPCT of 74 patients used variable dosing to achieve a target serum level of 400–600 ng/mL. This level is slightly higher than that typically used after solid organ transplant (100–400 ng/mL). A slower rate of progression was seen in men who started to receive treatment within 18 months of symptom onset. However, those who entered the study later in the disease course than 18 months showed no improvement (69).

### Total Lymphoid Irradiation

Irradiating the thymus, spleen and lymph nodes (while shielding non-lymphoid organs) is a treatment used typically in preparation for organ transplant and occasionally for severe cases of autoimmune diseases such as RA. One RDBPCT with 61 patients showed no difference, after 2 years observation, in muscle strength (*via* MRC or dynamometer), swallowing (time to swallow 4 oz water), walking (time to walk 15 and 25 feet) or survival (70).

### Prevention of Transplant Rejection

A variety of agents, typically in combination, are used to prevent acute graft-versus-host disease in transplant recipients. An observation in ALS patients undergoing neural stem cell injections into the spinal cord lead to the hypothesis that the immune-suppression required for the injections was, at least in part, responsible for the therapeutic effects. A Phase 2 trial with 33 patients to assess this has been completed (NCT01884571). This used basiliximab (anti-IL2) with mycophenolate (an inhibitor of nucleotide synthesis, which T- and B-cells rely upon) and tacrolimus (inhibits translocation of NFATC1 and thus transcription of IL2, TNFA, IFNG and NOS2 among other effects) as well as steroids.

## Discussion

The majority of the agents we reviewed appear safe and well tolerated; many are already in use for other conditions and so good information is available regarding side effects and monitoring. The most promising approaches identified in our review included NP001, masitinib–riluzole, celecoxib–creatine and tamoxifen–creatine [(58, 66, 71), NCT02588677]. All of these treatments await confirmation in larger RCTs.

As ALS progresses, so does the risk of infection, particularly aspiration pneumonia. Also, the part played by ‘tractable/modifiable inflammation’ relative to ‘degeneration’, as a cause of clinical disability, is likely to decrease, although this requires further investigation. In the case of secondary-progressive MS, where this transition has been better studied, CNS inflammation does not appear to cease, but becomes more difficult to address as much of the inflammation it is ‘hidden’ behind the BBB (72).

Thus, the risk/benefit ratio to immunosuppression in ALS is likely to increase with disease progression. A number of the treatments above are strongly immune-suppressing and the risk of infection associated with such medicines is well recognized in treating other autoimmune disorders, particularly in rheumatology (71). The risk of cancer with long-term immunosuppression in the setting of solid organ transplant has also been acknowledged. With a follow-up of 15 years after transplant, the sex- and age-standardized incidence ratio (i.e., number of cancers observed/number of cancers expected) has been estimated at 2.2 (73).

In other inflammatory conditions with progressive disability, patients appear willing to accept an early risk of complications in return for preventing later disability. In the case of a survey of MS patients, in return for approximately 5 years free of relapse and disability, patients were willing to accept a risk of approximately 1% for each of progressive multifocal leukoencephalopathy, leukemia and liver failure (74). Similar patient risk/benefit preferences have not been studied for ALS. Such work will likely need to wait for robust estimates for a treatment with proven efficacy.

The immune-suppressing treatment with the greatest potential short-term risk and arguably the greatest prospect of long-term control remains autologous bone marrow transplantation (ABMT). Preliminary experience of safety and efficacy in MS has proved encouraging, with transplant-related mortality rates of 0.3% (349 patients transplanted after 2005) and 0% (119 patients treated with a low-intensity regimen) (75). ABMT is not under investigation in ALS, to our knowledge.

Many of the drugs discussed above have also proved effective in psoriasis, a common inflammatory condition that also injures ectoderm-derived cells and is characterized by a chronic T_h_1 response, but that has received almost no attention in relation to ALS. While psoriasis tends to develop at a far younger age, any association (or lack thereof) in an individual or family affected by ALS could improve our understanding of both conditions. Of course, affecting inflammation within the relatively ‘immune-privileged’ CNS (vs. skin) has proved to be more challenging, in part due to accessibility and differences in pharmacokinetics.

The problem in translating work in mice into trials in humans in ALS is well recognized and has been reviewed by Mitsumoto et al. (5). SOD1-G93A remains the predominant mouse model. Given the rarity of SOD1-G93A as a cause of fLAS, trials conducted in those affected have proved difficult to perform and arguably would be limited relevance to sALS. Importantly, this mouse model has a different cause than sALS; the latter appears to have a variety of predisposing and precipitating factors, all of which converge on motor neuron death. This phenomenon, whereby a variety of pathological processes converge on a common phenotype, is also seen in Parkinson’s and (arguably) in Alzheimer’s disease. In both conditions, a subtype characterized by inflammation (vs. other metabolic abnormalities) has been proposed (76, 77). Splitting or stratifying such ‘hot’ vs. ‘cold’ disease subtypes appears to be a rational strategy, particularly while investigating anti-inflammatory and immune-suppressing treatments.

Focusing on the mediators of disease progression (vs. inciting mechanisms), particularly the immune system, thus offers the promise of treatments which are applicable to a wide range of patients with ALS.

Despite these challenges and the number of negative results, work by trialists has done much to improve our understanding of ALS in untreated patients. The ALS Patient Care Database should also prove valuable in this regard (78).

Whether ALS may be said to have a ‘natural history’ is debatable, as continuous improvements in supportive care have led to better outcomes for those affected. This is particularly true in the case of preventing infections, pressure ulcers, malnutrition (with feeding *via* gastrostomy if necessary) and respiratory failure (with non-invasive ventilation). Bearing this in mind, we hope that a ‘pooled’ cohort of patients receiving placebo as part of a clinical trial will become available. Such patients could serve as historical controls for new treatments in early stages of development. However, the quality of supportive care would need to be accounted for in such a pooled group; as a surrogate, the date and location of those receiving placebo on trial could be used.

## Conclusion

A number of basic science and clinical research studies have demonstrated a correlation between the immune system and ALS pathology. Despite these findings, the optimal targets have yet to be elucidated. It remains to be seen if inflammation is of similar importance in all forms of ALS. Improving our knowledge of inflammatory biomarkers that correlate with ALS progression may speed the development of such immune-modulating treatments.

## Author Contributions

SK, LA and RK all contributed to drafting the manuscript, reviewing references, and generating table and figures. SL provided helpful suggestions for improvement. CD prepared the final version of the manuscript, table and figures. All the authors have read and approved the final version.

## Conflict of Interest Statement

The authors declare that the research was conducted in the absence of any commercial or financial relationships that could be construed as a potential conflict of interest.

## Footnotes

^1^http://vimeo.com/66738855.
